# Plasma Amino Acids Metabolomics' Important in Glucose Management in Type 2 Diabetes

**DOI:** 10.3389/fphar.2021.695418

**Published:** 2021-07-15

**Authors:** Abdelrahim Alqudah, Mohammed Wedyan, Esam Qnais, Hassan Jawarneh, Lana McClements

**Affiliations:** ^1^ Department of Clinical Pharmacy and Pharmacy Practice, Faculty of Pharmaceutical Sciences, The Hashemite University, Zarqa, Jordan; ^2^ Department of Biology and Biotechnology, Faculty of Science, The Hashemite University, Zarqa, Jordan; ^3^ School of Life Sciences, Faculty of Science, University of Technology Sydney, Sydney, NSW, Australia

**Keywords:** type 2 diabetes, amino acids, metabolism, glucose, lipids

## Abstract

The perturbation in plasma free amino acid metabolome has been observed previously in diabetes mellitus, and is associated with insulin resistance as well as the onset of cardiovascular disease in this population. In this study, we investigated, for the first time, changes in the amino acid profile in a group of people with and without type 2 diabetes (T2D) with normal BMI, from Jordan, who were only managed on metformin. Twenty one amino acids were evaluated in plasma samples from 124 people with T2D and 67 healthy controls, matched for age, gender and BMI, using amino acids analyser. Total amino acids, essential amino acids, non-essential amino acids and semi-essential amino acids were similar in T2D compared to healthy controls. Plasma concentrations of four essential amino acids were increased in the presence of T2D (Leucine, *p* < 0.01, Lysine, *p* < 0.001, Phenylalanine, *p* < 0.01, Tryptophan, *p* < 0.05). On the other hand, in relation to non-essential amino acids, Alanine and Serine were reduced in T2D (*p* < 0.01, *p* < 0.001, respectively), whereas Aspartate and Glutamate were increased in T2D compared to healthy controls (*p* < 0.001, *p* < 0.01, respectively). A semi-essential amino acid, Cystine, was also increased in T2D compared to healthy controls (*p* < 0.01). Citrulline, a metabolic indicator amino acid, demonstrated lower plasma concentration in T2D compared to healthy controls (*p* < 0.01). These amino acids were also correlated with fasting blood glucose and HbA1c (*p* < 0.05). Glutamate, glycine and arginine were correlated with the duration of metformin treatment (*p* < 0.05). No amino acid was correlated with lipid profiles. Disturbances in the metabolism of these amino acids are closely implicated in the pathogenesis of T2D and associated cardiovascular disease. Therefore, these perturbed amino acids could be explored as therapeutic targets to improve T2D management and prevent associated cardiovascular complications.

## Introduction

According to the World Health Organisation, the number of people with diabetes increased from 108 million in 1980 to 422 million in 2014, and the prevalence of diabetes has been rising rapidly in the middle- and low-income countries (WHO, 2014). In 2016, approximately 1.6 million deaths were directly attributed to diabetes, with half of all deaths occurring before the age of 70 (WHO, 2014). In 2004, the prevalence of diabetes among Jordanian population aged between 25 and 70 was 17.1%, increasing to 23.7% in 2017, which is significantly higher than the world average. This increase in the prevalence of diabetes in Jordan is likely due to poor diet and a more sedentary lifestyle (Ajlouni et al., 2019). Diabetes is a metabolic disorder characterised by chronic hyperglycaemia, affecting carbohydrate, fat, and protein metabolism leading to abnormal insulin secretion, insulin resistance, or both (Silva et al., 2018). Diabetes mellitus is characterised by the following symptoms: thirst, polyuria, blurred vision, and weight loss. These symptoms can be quiescent for a prolonged period of time resulting in retinopathy, nephropathy, neuropathy and cardiovascular complications due to persistent hyperglycaemia, occurring before the diagnosis of diabetes mellitus is established (WHO, 1999). The most frequent form of diabetes is type 2 diabetes (T2D). T2D is characterised by hyperglycaemia, insulin resistance and relative insulin deficiency, often at later stages in life (Chatterjee, Khunti and Davies, 2017). The causes of T2D are still not fully understood; however, high body mass index (BMI), age, ethnicity and family history have all been linked to the epidemiology of T2D (Schwarz et al., 2009). Other factors have also shown some association with diabetes including poor diet and nutrition, physical inactivity, impaired glucose tolerance, smoking, and past history of exposure of the unborn child to high glucose during pregnancy (maternal gestational diabetes) (Feig and Palda, 2002). Furthermore, recent evidence has suggested that high sugar intake is associated with the risk of T2D (Malik et al., 2010).

Amino acids (AA) are the building units of proteins with critical roles in gene expression, cell signalling, reproduction, neurotransmission, metabolism, oxidative stress, pain control, inflammatory responses, and detoxification (Al-Abbasi, 2013). Human cells can synthesize several amino acids including alanine (Ala), asparagine (Asn), aspartate (Asp), glutamate (Glu), glutamine (Gln), glycine (Gly), proline (Pro) and serine (Ser) that are referred to as non-essential amino acids. On the other hand, some amino acids are obtained from the diet and referred to as essential amino acids. These include histidine (His), isoleucine (ILe), leucine (Leu), lysine (Lys), methionine (Met), phenylalanine (Phe), threonine (Thr), tryptophan (Trp), and valine (Val). Semi-essential amino acids including arginine, cysteine (Cys), and tyrosine (Tyr) can be synthesised endogenously, however in insufficient amounts, therefore, these are required partially to be obtained from the diet (Cunnane, 2003). Other amino acids with the metabolism conversion capacity or dietary adequacy have a role as metabolic indicators of impaired metabolism and these include ornithine (Orn) and citrulline (Cit) (Al-Abbasi, 2012). Metabolomics investigations have found a strong association between some amino acids and insulin resistance (Newgard, 2012). Furthermore, a number of published studies reported that certain pathological conditions including metabolic and cardiovascular diseases are associated with disturbances in plasma free amino acids (PFAA) metabolism (Shah, Kraus and Newgard, 2012), hence these amino acids could be utilised as potential target for therapeutic intervention in such conditions including diabetes. Disturbances in amino acid regulation in diabetes were first reported decades ago were Leu, ILe, and Val were increased, and Ala, Gly, Thr, and Ser were reduced in people with diabetic ketoacidosis, suggesting that the dysregulation of these amino acids could contribute to the development of acute diabetic syndrome (Felig et al., 1970). More recently, a prospective cohort study concluded that a higher dietary intake of Branched chain amino acids (BCAA) was associated with increased risk of T2D (Zheng et al., 2016). Using an untargeted metabolomics approach, certain BCAAs (Val, ILe and Leu) were significantly elevated in both individuals with impaired fasting glucose (IFG) or T2D compared to healthy controls (Menni et al., 2013). In addition, total and individual BCAAs levels were found to be elevated as a result of diabetes or high HbA1c and were positively associated with dyslipidaemia, suggesting that BCAAs could have a predictive potential for cardiovascular complications (Yang et al., 2016). Moreover, the alteration of amino acid profile was strongly correlated with visceral fat accumulation associated with the development of metabolic complications including insulin resistance and diabetes (Yamakado et al., 2012). A recent study revealed that alanine aminotransferase is increased in the liver of mice with obesity and diabetes, as well as in humans with T2D. Furthermore, silencing of alanine aminotransferase in hepatocytes in mice with obesity and diabetes abrogated hyperglycaemia and restored skeletal muscle protein synthesis improving insulin sensitivity, which suggests that liver aminotransferase has a key role in inducing skeletal muscle atrophy in T2D (Okun et al., 2021). Although, aberrant PFAA concentrations have been reported previously in people with T2D in different countries, no comprehensive study was conducted to assess the PFAA concentrations in T2D with normal BMI, in Jordanian cohorts, who were only taking metformin (Al-Abbasi, 2012; Yamaguchi et al., 2017). Therefore, this study aims to determine amino acid metabolic profile in non-obese undertreated people with T2D who were only taking metformin compared to healthy controls, providing a better understanding of the role of individual amino acids metabolism and metformin treatment in this study population.

## Methods

### Study Population

Participants with T2D or healthy controls referred to Endocrinology Department at the Princess Bassma public hospital and King Abdullah University hospital between January to April 2019, were fully informed about the study before providing the consent and donating a blood sample. A total of 124 participants with T2D and 67 healthy controls were recruited in this study. Inclusion criteria was set as follows: T2D diagnosed at least 6 months prior to sample collection, residing in Jordan, age >30 years old, and treatment regimen that included metformin only in addition to unstructured physical activity such as household tasks, walking, and gardening for three to 15 min after meals to increase daily energy expenditure and assist the weight management. The participants were only treated with metformin and refused addition treatment with other hypoglycaemic agents when recommended by their treating physician due to fear of side effects. Exclusion criteria included the following conditions: type 1 diabetes and chronic complications of T2D (nephropathy, retinopathy, and cardiovascular disease).

Ethical approval for this study was obtained from the Institutional Review Board (IRB number 6/7/2017/2018) at the Hashemite University, Zarqa, Jordan, and all procedures followed were in accordance with the Declaration of Helsinki.

### Clinical and Laboratory Assessments

T2D was diagnosed according to the American Diabetes Association criteria (American Diabetes Association, 2018), which was based on fasting blood glucose (FBG) ≥ 126 mg/dl after no calory intake for at least 8 h. The absence of diabetes in the control group was confirmed through assessing the FBG on two separate occasions. BMI was calculated using self-reported weight (kg) divided by self-reported height in meters squared (kg/m^2^). Blood pressure was measured by trained nurses 3 times using the right arm after a 10 min rest period. Venous blood was collected from peripheral vein in heparinized collection tubes. Blood samples were mixed gently, centrifuged at 3,000 rpm at 4°C for 15 min before the plasma was collected and stored at −80°C for further analysis.

### Fasting Blood Glucose and HbA1c Analysis

HbA1c values were measured using commercially available kit (Wondfo, China, cat#: W207)) and blood glucose levels were assessed using colorimetric detection kit (Biolabo, France, cat# 80009) according to the manufacturer’s instructions.

### Lipid Profile Analysis

Total cholesterol and high density lipoproteins (HDL) were analysed using commercially available kit (Biolabo, France, cat#: 80106, 86516, respectively) according to the manufacturer’s instructions. Triglycerides (TGs) were measured using commercially available kit (Linear Chemicals, Spain, cat#: 1155005) according to the manufacturer’s instructions. Low density lipoproteins (LDL) were calculated according to the following formula: LDL = Total cholesterol-HDL-(TG/5) (Friedewald, Levy and Fredrickson, 1972)

### Amino Acid Analysis

In order to reduce bias with data analysis, samples were given a unique code and were blinded. Plasma samples were prepared and deproteinized using amino acid sample preparation kit (MembraPure Gmbh, Germany, version ORRaK) according to the manufacturer’s instructions, which removes proteins using precipitation solution followed by 0.22 µm filtration. Molar concentration of salts in the sample was maintained below the level that interferes with the chromatographic separation. In addition, the pH of the sample was controlled around 2.2 to avoid negative impact on chromatographic separation. All sample preparation was performed at 4°C to prevent heat liable amino acids from degradation. Total amino acid profiling was carried out using Amino Acid Analyzer (ARACUS, version 2.0, membraPure Gmbh, Germany).

### Statistical Analysis

Data were represented as mean ± SD. Prism 5 software (Graphpad software, United States) was used for the analysis of the data. All analysed parameters were tested for normality of the data using the Kolmogorov-Smirnov test. Unpaired student t-test was used to examine the difference in plasma amino acids concentration between the tested groups. Pearson’s correlation test was used to determine the correlation between plasma amino acids concentration and FBG, HbA1c, length of metformin treatment, lipid profile, and blood pressure. *p* < 0.05 was taken as the cut off value for statistical significance.

## Results

### Baseline Characteristics

Plasma samples were collected from 124 non-obese people with T2D, with the average diagnosis time of 10.5 years and the average duration of metformin treatment of 9.1 years, and 64 samples from healthy controls. Baseline characteristics are shown in Table 1. No statistically significant differences in age, gender, BMI, systolic and diastolic blood pressure, cholesterol, and HDL were noted between the study group and controls. As expected, people with T2D had higher blood concentration of FBG and HbA1c compared to healthy controls (Table 1, *p* = 0.001). LDL and TG levels were higher in people with T2D compared to healthy controls (Table 1, *p* < 0.002, 0.001, respectively).

**TABLE 1:** Clinical characteristics for the participants.

Clinical characteristics	Control (*n* = 67)	T2D (*n* = 124)	*p* value
Age (year)	64.1 ± 6.9	62.6 ± 12	0.33
Gender	35 males	75 males	0.27
	32 females	49 females	
BMI (Kg/m^2^)	23.1 ± 2.4	23.9 ± 3.1	0.10
Systolic Blood Pressure (mmHg)	121.6 ± 2.9	124.1 ± 11.4	0.08
Diastolic Blood Pressure (mmHg)	79 ± 3.9	80.8 ± 8.9	0.11
HbA1c (mmol/mol)	38 ± 4	74 ± 15	**0.001**
HbA1c (%)	5.6 ± 0.3	8.9 ± 1	**0.001**
FBG (mg/dl)	113 ± 10.5	212 ± 41.6	**0.001**
Cholesterol (mg/dl)	152.6 ± 26.5	160 ± 31.7	0.10
HDL (mg/dl)	49.1 ± 7.0	50.7 ± 7.2	0.15
LDL (mg/dl)	73.5 ± 32	89.5 ± 33.0	**0.002**
Triglyceride (mg/dl)	85.2 ± 34.1	99.3 ± 28.7	**0.001**

The bold values indicate statistically significant values.

### Plasma Levels of the Main Groups of Amino Acids in People With T2D

**FIGURE 1:**
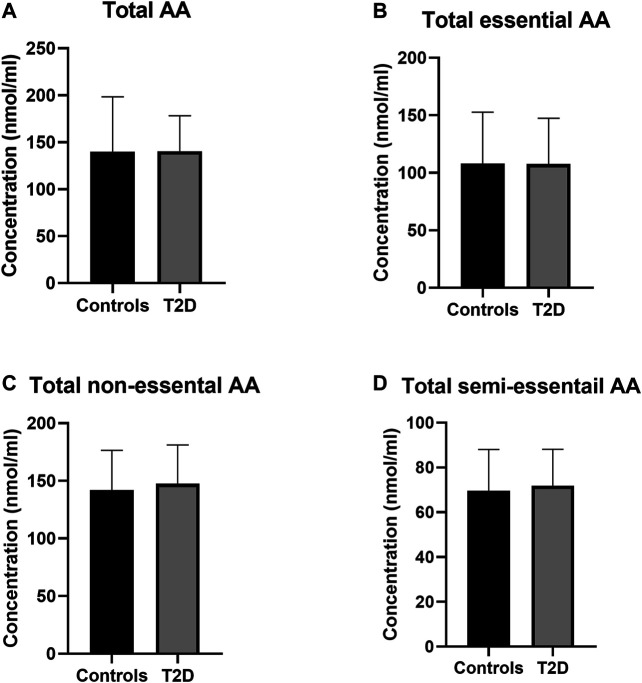
Plasma concentration of total amino acids in type 2 diabetes (T2D). No significant difference in free plasma concentration of total amino acids **(A)**, essential amino acids **(B)**, non-essential amino acids **(C)**, and semi-essential amino acids **(D)** between people with T2D and healthy controls. Plasma samples from T2D people (*n* = 124) or healthy controls (*n* = 67) were prepared and deproteinized before total amino acid profiling was carried out (unpaired independent *t*-test).

The total PFAA concentrations were assessed in order to determine any disturbances in amino acid metabolism in this group of Jordanian participants as a result of T2D. No statistically significant difference was observed in the total amino acids concentrations between T2D participants and healthy controls (Figure 1A). Also, total essential, non-essential and semi-essential PFAA concentrations were not statistically different between these two groups (Figures 1B–D respectively).

### Regulation of Essential Amino Acids in T2D

**FIGURE 2:**
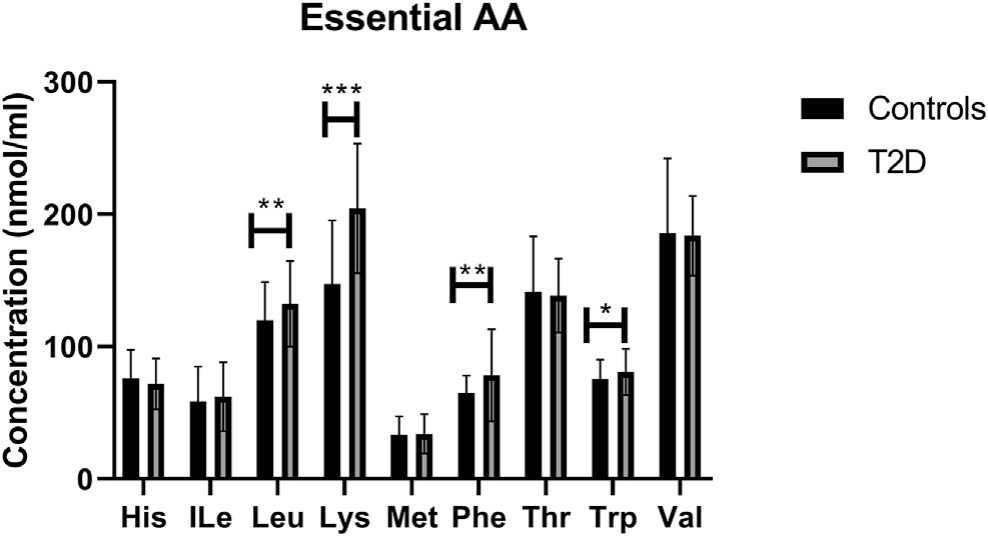
Plasma concentration of essential amino acids in type 2 diabetes (T2D). Statistically significant increase in Leu, Lys, Phe, and Trp was demonstrated between people with T2D and healthy controls. Plasma samples from T2D participants (*n* = 124) or healthy controls (*n* = 67) were prepared and deproteinized before total amino acid profiling was carried out (unpaired independent *t*-test, *<0.05, **<0.01).

By analysing individual essential PFAA concentration, no significant difference in free plasma concentrations of His, ILe, Met, Thr, and Val was found between people with T2D and controls (Figure 2). Furthermore, no significant correlation between these amino acids and FBG or HbA1c was observed in T2D (FBG, Table 2; HbA1c, Table 3). However, significant increase in free plasma concentrations of Leu, Lys, Phe and Trp was noted in people with T2D compared to healthy controls (Figure 2; Leu, *p* <0.01, Lys, *p* <0.001, Phe, *p* <0.01, Trp, *p* <0.05). These results were consistent when correlation was performed between essential amino acids and FBG. Leu, Lys, Phe, and Trp were positively correlated with FBG (Table 2; Leu, *r* = 0.53, *p* = 0.004; Lys, *r* = 0.395, *p* = 0.03; Phe, *r* = 0.42, *p* = 0.006; Trp, *r* = 0.675, *p* = 0.0001). Similarly, Leu, Lys, Phe, and Trp were positively correlated with HbA1c (Table 3; Leu, *r* = 0.44, *p* = 0.02; Lys, *r* = 0.43, *p* = 0.012; Phe, *r* = 0.49, *p* = 0.001; Trp, *r* = 0.59, *p* = 0.001). No significant correlation was found between essential amino acids and the length of metformin treatment (Table 4), lipid profile (Table 5) and blood pressure (Table 6).

**TABLE 2:** Correlation analysis between FBG and amino acids concentrations in T2D.

Amino acid	Correlation (r)	*p* value
His	0.050	0.80
ILe	0.164	0.41
Leu	0.53	**0.004**
Lys	0.395	**0.03**
Met	−0.161	0.42
Phe	0.42	**0.006**
Thr	−0.163	0.18
Trp	0.675	**0.0001**
Val	−0158	0.58
Ala	−0.46	**0.01**
Asn	−0.019	0.88
Asp	0.886	**0.001**
Glu	0.550	**0.001**
Gln	−0.041	0.87
Gly	−0.178	0.24
Ser	−0.555	**0.0004**
Arg	−0.365	0.09
Cys	0.569	**0.005**
Tyr	−0.261	0.14
Orn	0.312	0.08
Cit	−0.438	**0.01**

The bold values indicate statistically significant values.

**TABLE 3:** Correlation analysis between HbA1c and amino acids concentrations in T2D.

Amino acid	Correlation (r)	*p* value
His	0.076	0.69
ILe	0.104	0.56
Leu	0.44	**0.02**
Lys	0.43	**0.012**
Met	0.055	0.74
Phe	0.49	**0.001**
Thr	−0.083	0.69
Trp	0.59	**0.001**
Val	0.069	0.81
Ala	−0.6	**0.0001**
Asn	−0.11	0.45
Asp	0.69	**0.04**
Glu	0.53	**0.001**
Gln	0.156	0.52
Gly	−0.032	0.87
Ser	−0.35	**0.03**
Arg	−0.308	0.08
Cys	0.79	**0.0007**
Tyr	−0167	0.30
Orn	0.118	0.52
Cit	−0.51	**0.003**

The bold values indicate statistically significant values.

**TABLE 4:** Correlation analysis between duration of metformin treatment and amino acids concentrations in T2D.

Amino acid	Correlation (r)	*p* value
His	−0.04	0.79
ILe	−0.09	0.61
Leu	−0.02	0.91
Lys	−0.24	0.27
Met	0.15	0.36
Phe	0.30	0.06
Thr	−0.27	0.19
Trp	−0.24	0.23
Val	0.06	0.82
Ala	0.04	0.82
Asn	0.04	0.79
Asp	0.35	0.36
Glu	0.55	**0.01**
Gln	−0.17	0.48
Gly	−0.43	**0.02**
Ser	−0.25	0.13
Arg	0.59	**0.004**
Cys	−0.09	0.77
Tyr	−0.01	0.96
Orn	−0.30	0.20
Cit	−0.03	0.87

The bold values indicate statistically significant values.

**TABLE 5:** Correlation analysis between lipid profile and amino acids concentrations in T2D.

Amino acid	Cholesterol		TG		LDL		HDL	
	Correlation (r)	*p* value	Correlation (r)	*p* value	Correlation (r)	*p* value	Correlation (r)	*p* value
His	−0.22	0.89	0.04	0.80	−0.01	0.97	−0.10	0.54
ILe	−0.04	0.81	0.11	0.56	0.02	0.92	−0.33	0.06
Leu	−0.19	0.33	−0.13	0.51	−0.21	0.29	0.21	0.29
Lys	0.03	0.91	−0.27	0.22	0.06	0.78	0.04	0.86
Met	−0.02	0.90	−0.19	0.26	0.02	0.91	−0.03	0.87
Phe	−0.05	0.76	−0.17	0.29	−0.03	0.86	0.05	0.76
Thr	0.19	0.35	0.21	0.30	0.24	0.25	−0.36	0.07
Trp	0.05	0.82	0.12	0.55	0.11	0.57	−0.34	0.08
Val	0.06	0.83	0.22	0.44	0.06	0.82	−0.17	0.55
Ala	−0.03	0.88	0.04	0.83	0.02	0.92	−0.20	0.30
Asn	0.24	0.10	−0.16	0.29	0.24	0.11	0.07	0.64
Asp	−0.43	0.25	−0.25	0.52	−0.47	0.20	0.38	0.32
Glu	−0.05	0.80	−0.10	0.59	−0.05	0.79	0.10	0.59
Gln	0.20	0.41	−0.28	0.25	0.21	0.40	0.11	0.67
Gly	−0.15	0.42	−0.11	0.56	−0.13	0.50	0.04	0.85
Ser	−0.14	0.42	0.08	0.62	−0.17	0.32	0.12	0.48
Arg	0.07	0.77	0.07	0.76	0.01	0.96	0.11	0.62
Cys	−0.02	0.95	0.21	0.46	0.11	0.71	−0.44	0.11
Tyr	0.23	0.18	−0.24	0.15	0.26	0.12	−0.04	0.82
Orn	0.15	0.54	−0.22	0.35	0.17	0.47	0.03	0.90
Cit	0.04	0.83	0.27	0.13	−0.00	0.99	−0.06	0.76

**TABLE 6:** Correlation analysis between blood pressure and amino acids concentrations in T2D.

Amino acid	Correlation (r) with SBP	*p* value	Correlation (r) with DBP	*p* value
His	0.074	0.69	0.259	0.16
ILe	0.229	0.13	0.270	0.072
Leu	−0.049	0.81	0.149	0.45
Lys	−0.065	0.73	0.117	0.54
Met	0.028	0.86	−0.199	0.22
Phe	0.158	0.33	0.011	0.94
Thr	0.138	0.25	0.026	0.82
Trp	−0.019	0.92	0.196	0.32
Val	0.134	0.63	−0.23	0.40
Ala	−0.083	0.66	0.126	0.50
Asn	0.049	0.68	0.003	0.97
Asp	0.331	0.38	0.221	0.33
Glu	−0.039	0.83	−0.137	0.46
Gln	0.293	0.22	0.197	0.41
Gly	−0.130	0.38	0.197	0.18
Ser	−0.046	0.78	0.119	0.48
Arg	0.219	0.32	0.148	0.50
Cys	0.028	0.90	0.066	0.76
Tyr	0.057	0.72	0.108	0.49
Orn	−0.098	0.59	−0.069	0.70
Cit	−0.105	0.56	−0.008	0.96

### Regulation of Non-essential Amino Acids in T2D

**FIGURE 3:**
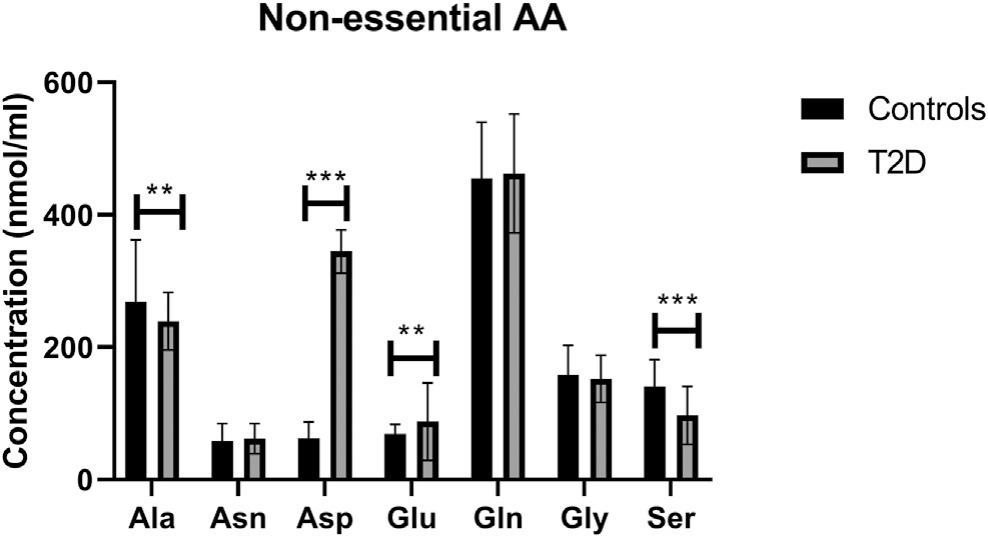
Plasma concentration of non-essential amino acids in type 2 diabetes (T2D). Ala and Ser concentration was reduced in T2D, whereas Asp and Glu concentrations were increased compared to healthy controls. Plasma samples from T2D participants (*n* = 124) or healthy controls (*n* = 67) were prepared and deproteinized before total amino acid profiling was carried out (unpaired independent *t*-test, **<0.01, ***<0.001).

In terms of the non-essential amino acids, no statistically significant difference was noted in free plasma concentrations of Asn, Gln, and Gly between T2D and control groups (Figure 3); the correlation between free plasma concentrations of these amino acids and FBG or HbA1c in T2D was also not statistically significant (Table 2 and Table 3, respectively). Plasma levels of Ala and Ser were significantly decreased in people with T2D compared to healthy controls (Figure 3, Ala, *p* <0.01, Ser, *p* <0.001), whereas, Asp and Glu showed a significant increase in people with T2D compared to healthy controls (Figure 3; Asp, *p* <0.001, Glu, *p* <0.01). Similarly, Ala and Ser plasma concentrations in people with T2D were negatively correlated with FBG (Table 2; Ala, *r* = −0.46, *p* = 0.01; Ser, *r* = −0.555, *p* = 0.0004). In addition, HbA1c levels were negatively correlated with Ala and Ser levels (Table 3; Ala, *r* = −0.6, *p* = 0.0001; Ser, *r* = −0.35, *p* = 0.03), whilst Asp and Glu concentrations were positively correlated with FBG (Table 2; Asp, *r* = 0.886, *p* = 0.001; Glu, *r* = 0.55, *p* = 0.001) and HbA1c levels (Table 3; Asp, *r* = 0.69, *p* = 0.04; Glu, *r* = 0.53, *p* = 0.001) in T2D. In addition, Glu was found to have a positive correlation with the length of metformin treatment, whereas, Gly was found to have a negative correlation with metformin length of treatment (Table 4; Glu, *r* = 0.55, *p* = 0.01; Gly, *r* = −0.43, *p* = 0.02). No significant correlation was found between non-essential amino acids and lipid profile (Table 5) and blood pressure (Table 6).

### Regulation of Semi-essential and Metabolic Indicators Amino Acids in T2D

**FIGURE 4:**
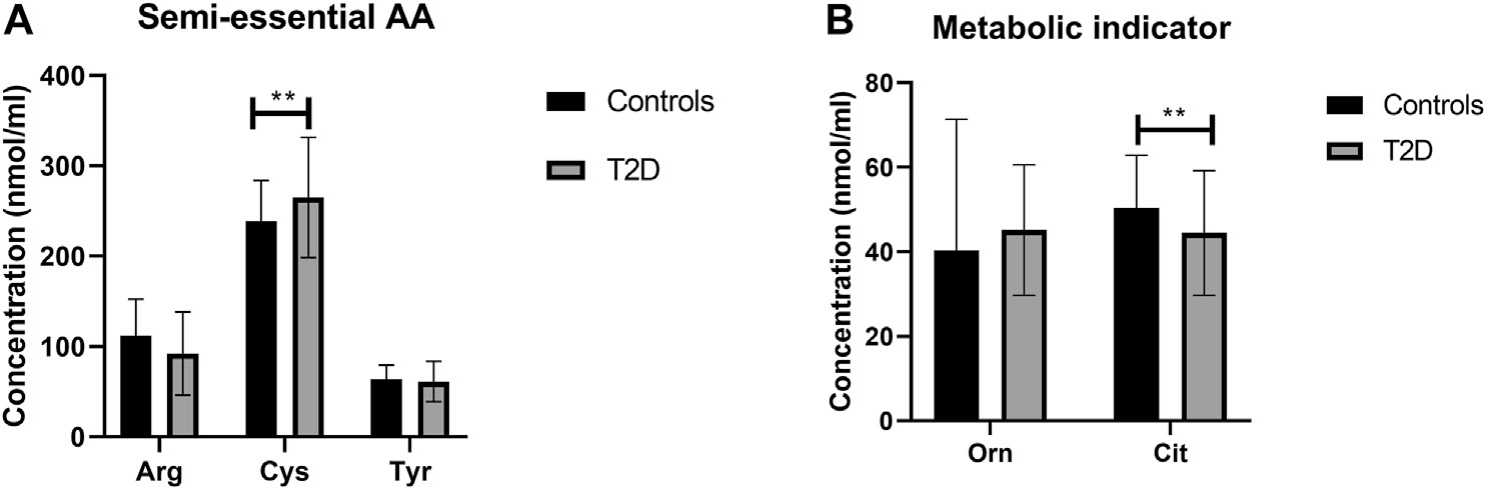
Plasma concentration of semi-essential **(A)** and metabolic indicators **(B)** amino acids in type 2 diabetes (T2D). Cys plasma concentration was increased in T2D participants compared to healthy controls **(A)**. Cit plasma concentration was reduced in T2D compared to healthy control **(B)**. Plasma samples from T2D participants (*n* = 124) or healthy controls (*n* = 67) were prepared and deproteinized before total amino acid profiling was carried out (unpaired independent *t*-test, **<0.01).

There was no statistically significant difference in semi-essential amino acids, Arg and Tyr, between T2D and healthy controls (Figure 4A). In addition, the correlation between these amino acids and FBG (Table 2) or HbA1c (Table 3) in T2D was not demonstrated (Table 2). However, Cys was significant increased in people with T2D compared to healthy controls (Figure 4A, *p* <0.01). Moreover, Cys free plasma concentration was positively correlated with FBG (Table 1; *r* = 0.569, *p* = 0.005) and HbA1c (Table 3; *r* = 0.79, *p* = 0.0007) levels in T2D. Furthermore, Arg showed a positive correlation with the length of metformin treatment (Table 4; *r* = 0.59, *p* = 0.004). No significant correlation was found between semi-essential amino acids and lipid profile (Table 5) or blood pressure (Table 6).

With regards to metabolic indicators amino acids, Orn free plasma concentration did not show a significant difference between people with T2D and controls (Figure 4B), and the correlation between Orn and FBG (Table 2) or HbA1c (Table 3) in T2D was not statistically significant. Interestingly, Cit was significantly decreased in people with T2D compared to healthy controls (Figure 4B, *p* <001). In addition, Cit free plasma concentration was negatively correlated with FBG (Table 2; *r* = −0.438, *p* = 0.01) and HbA1c (Table 3; *r* = −0.51, *p* = 0.003) levels in T2D. No significant correlation was found between metabolic indicators amino acids and lipid profile (Table 5) or blood pressure (Table 6).

## Discussion

To the best of our knowledge, this is the first study describing the association between T2D and PFAA concentrations in non-obese Jordanian participants treated with metformin only. This study sheds further light on the regulation of specific amino acid in T2D and association with metabolic profile and metformin treatment in this study population. A recent study conducted in China reported that there was a significant increase in plasma concentration of six essential amino acids (ILe, Leu, Lys, Phe, Trp and Val), three non-essential and semi-essential amino acids (Ala, Glu and Tyr) in T2D, and this increase was associated with higher risk of T2D prevalence and/or incidence (Lu et al., 2019). Aligned to the findings of this study, here we demonstrate that Leu, Lys, Phe, Trp, and Glu plasma concentrations were significantly increased in people with T2D compared to controls, and positively associated with poor glucose management. Furthermore, some reports suggested that essential amino acids including BCAA, ILe, Leu, and Val, obtained from the diet and metabolised in adipose tissues and skeletal muscles, are strongly associated with insulin resistance (Chen et al., 2016). Moreover, it has been previously demonstrated that BCAA were higher in T2D, and this elevation was positively correlated with insulin resistance. However, these findings were not attributed to leptin and adiponectin, major contributors of insulin resistance, suggesting that BCAA could induce insulin resistance via a different mechanism (Connelly, Wolak-Dinsmore and Dullaart, 2017). Our results demonstrated that Leu plasma concentration was significantly increased in people with T2D reflective of poor glucose management. Low levels of Leu were reported to improve insulin sensitivity in the liver by activating general control non-derepressible (GCN)2 and decreasing the activity of rapamycin/S6K1 signalling, in addition to AMPK activation, suggesting that high levels of Leu are strongly associated with insulin resistance (Xiao et al., 2011). The lack of energy intake and inactivity was found to reduce skeletal muscle mass leading to a reduction in muscle function and insulin resistance. Also, insulin resistance appears to drive skeletal muscle atrophy. Exercise and essential amino acids including Leu can stimulate human protein muscle synthesis and growth (Drummond et al., 2009; Rudrappa et al., 2016). In our study, Leu was found to be increased in people with T2D, thus, combining exercise with this increase in Leu level might be an essential strategy for those people to promote muscle protein synthesis hence improving insulin sensitivity. Surprisingly, our participants with T2D had normal BMI, which is not frequently observed in people with T2D. This could be due to the dysregulation of BCAA. Newgard and colleagues reported using a high-fat diet (HFD) animal model that supplementation with BCAA (HF/BCAA) reduced food intake and body weight without improving insulin resistance. This suggests that, the dysregulation of BCAA metabolism following HFD has an independent contribution to the development of obesity-associated insulin resistance (Newgard et al., 2009; Morris et al., 2012). In our study, Phe, an aromatic amino acid (AAA), was increased in T2D, which is consistent with several previously published studies showing that Phe concentration was elevated in response to obesity and insulin resistance (Wang et al., 2011); although our T2D participants were not obese. Moreover, our results showed that plasma concentration of another AAA, Trp, was also increased in T2D. Trp is implicated in tryptophan-kynurenine and tryptophan-methoxyindole metabolic pathways involved in the production of a number of active metabolites including kynurenine, kynurenic acid and serotonin. Any disturbances in this metabolic pathways are likely associated with T2D pathogenesis (Unluturk and Erbas, 2015). Furthermore, we also showed that Lys, an essential amino acid, was increased in people with T2D. Razquin et al., demonstrated that Lys levels is associated with higher risk of T2D. In addition, people with T2D and high levels of Lys appear to have increased risk of cardiovascular disease (Razquin et al., 2019).

On the other hand, Ala, a non-essential amino acid, was reported to be increased in hyperinsulinemia conditions in diabetes, and supplementation of Ala improved glucagon response to hypoglycaemia events in diabetes (Porcellati et al., 2007). Here, Ala plasma concentration was significantly reduced in T2D people, suggesting that the participants in this study might be at higher risk of hypoglycaemic events, which could be linked to their poorly managed blood glucose. Furthermore, our results showed that plasma concentrations of Asp and Glu, acidic amino acids, were increased in people with T2D, which is in line with previous reports showing that acidic amino acids plasma concentration was increased in T2D compared to controls (Cheng et al., 2012). Recent study reported that adiponectin concentration is negatively correlated with Asp and Glu concentrations with a potential to increase the risk of cardiovascular disease (Ntzouvani et al., 2017). Additionally, our results indicate that Ser, a non-essential amino acid, plasma concentration was significantly decreased in T2D. These results were consistent with a Japanese’s study reporting that a cohort who developed diabetes within four years of a follow-up period had a lower Ser concentration, which was closely associated with the development of metabolic syndrome (Yamakado et al., 2015).

In our study, Cys, a semi-essential amino acid, was elevated in T2D compared to controls and associated with high HbA1c. Previous reports show that Cys plasma concentration was positively correlated with obesity and insulin resistance, and that higher levels of Cys could lead to obesity (Elshorbagy et al., 2012). Moreover, higher concentration of Cys was positively correlated with markers of inflammation including C-reactive protein (CRP) and tumor necrosis factor-α (TNF-α), suggesting higher risk of diabetes complications (Mohorko et al., 2015). Cit is a key modulator of urea cycle in the liver and kidneys that can be synthesized from Orn by the intestines (Cynober, Boucher and Vasson, 1995). In addition, Cit can contribute to Arg synthesis before it is converted to nitric oxide (NO) by endothelial nitric oxide synthase (eNOS) (Flam, Eichler and Solomonson, 2007). NO has an essential role in maintaining endothelial function and vasodilation, in addition to regulating insulin sensitivity (Sansbury and Hill, 2014). Our results indicate a significant reduction in Cit free plasma concentration that could suggest that there is a reduction in NO and increased risk of future cardiovascular disease in these participants. Restoring Cit levels through supplementation could potentially prevent or delay cardiovascular complications in people with T2D.

Moreover, LDL and TGs were found to be significantly increased in people with T2D which is consistent with several previously published studies and also associated with the increased risk of cardiovascular complications (Khan, Sobki and Khan, 2007; Hirano, 2018). T2D is considered as a risk factor for non-alcoholic fatty liver disease (NAFLD), the most common cause of chronic liver disease and cardiovascular complications (Kalra et al., 2013; Zhang and Lu, 2015). A meta-analysis demonstrated that the prevalence of NAFLD among people with T2D was 55.5% (Younossi et al., 2019). Additionally, TG and LDL were showed to be higher in people with NAFLD with T2D compared to NAFLD without T2D (Shams, Al-Gayyar and Barakat, 2011). Insulin resistance facilitates the increase of free fatty acid flux leading to increased levels of LDL and TG, triggering oxidative stress and lipid peroxidation, all of which are closely associated with the development of NAFLD (Cao et al., 2013; Alkhouri et al., 2014). Our findings in this study indicate an elevation of LDL and TG, which could increase the incidence of NAFLD in our study population. As discussed before, most of the dysregulated amino acids in this study are associated with insulin resistance suggesting that participants with T2D in this study are at high risk of developing NAFLD. In addition, a previous study revealed that high Glu levels had a significant pathological role in the development of liver fibrosis independently from insulin resistance (Hasegawa et al., 2020). In this study, Glu level was significantly higher in T2D and together with increased TG and LDL could increase the risk of future liver fibrosis as well as cardiovascular complications.

In our T2D study population, who were only treated with metformin and displayed poorly managed glucose control, we identified three amino acids (Glu, Gly, Arg) that are potentially affected by the duration of metformin treatment. Previously published study showed that metformin administration for 6, 12, and 18 months decreases plasma levels of Phe and Tyr along with increased His and Ala plasma levels (Preiss et al., 2016). Another study showed that metformin administration increased plasma levels of Leu, ILe, and Tyr in T2D (Walford et al., 2013). In our study, interestingly a positive correlation was found between Glu plasma levels and the length of metformin treatment. Glu had a strong correlation with insulin resistance and the potential to increase the risk of cardiovascular disease (Seibert et al., 2015; Ntzouvani et al., 2017). On the other hand, the length of metformin treatment was positively correlated with Arg plasma concentration, which is a precursor for NO production that regulates insulin sensitivity (Flam, Eichler and Solomonson, 2007; Sansbury and Hill, 2014). Moreover, Gly, which was reported to have a positive association with insulin resistance (Lustgarten et al., 2013), in our study, was negatively correlated with the length of metformin treatment. Furthermore, metformin has the ability to increase protein synthesis in the muscles that could improve insulin sensitivity (Gore et al., 2005; He, 2020).

Interestingly, no significant correlation was found between systolic blood pressure/diastolic blood pressure (SBP/DBP) and any amino acid in our study population, which was consistent with several published studies. In one study, amino acids intake including Glu, Arg, Cys, Lys, and Tyr was not associated with the incidence of hypertension (Altorf-van der Kuil et al., 2013). Another study from China revealed that no significant correlation between either a single BCAA or total BCAAs and SBP/DBP was observed (Hu et al., 2016). On the other hand, people who had higher BCAAs dietary intake demonstrated lower SBP compared to those who had lower intake (Jennings et al., 2016). Conversely, Yamaguchi et al. showed a positive association between BCAAs and blood pressure (Yamaguchi et al., 2017). Furthermore, non-significant association between SBP and Arg higher intake was observed in the Dutch population (Oomen et al., 2000). In addition, Glu showed an independent inverse relation with blood pressure (Stamler et al., 2009). The results from the studies that evaluated the effect of amino acids on blood pressure are inconsistent and therefore need further investigation.

Limitations of this study include the following aspects of the study: 1) the levels of amino acids are partially influenced by the dietary habits, the information we did not obtain, 2) the sample size was small, cross-sectional and from Jordanian population only, hence limiting the application of our results, 3) body weight and height were self-reported, and 4) the information on NAFLD was not obtained in the study population. Nevertheless, our results aligned with the published literature warranting further investigations into abovementioned amino acids with impaired metabolism using larger longitudinal cohorts of people with T2D and healthy controls. Also, all perturbed amino acids were correlated in the same manner with FBG or HbA1c hence suggesting that these changes in amino acid profiles are reflective of blood glucose control.

In conclusion, this study demonstrated that specific amino acids with different important biological roles are dysregulated in non-obese undertreated people with T2D impacting on glucose management. The findings presented in this study suggest that dysregulated amino acids in T2D have the potential to increase the risk of future diabetic complications, and hence should be explored as therapeutic targets or corrected through supplementation to improve T2D management and prevent future complications.

## Data Availability

The datasets generated during and/or analysed during the current study are available from the corresponding author on reasonable request.
